# Conducting research on building psychosocial support for Syrian refugee families in a humanitarian emergency

**DOI:** 10.1186/s13031-021-00365-6

**Published:** 2021-04-23

**Authors:** Stevan Merill Weine, Aliriza Arënliu, Vahdet Görmez, Scott Lagenecker, Hakan Demirtas

**Affiliations:** 1 Center for Global Health, University of Illinois in Chicago, 1940 W. Taylor M/C 584, Chicago, IL 60612 USA; 2 Department of Psychology, Philosophical Faculty, University of Prishtina “Hasan Prishtina, Eqrem Çabej, nn., 10000 Prishtina, Kosovo; 3 Child and Adolescent Psychiatry, Medeniyet Universitesi, Dr. Erkin Cd, 34722 Kadıköy/İstanbul, Turkey; 4 University Neuropsychiatric Institute, 501 Chipeta Way, Salt Lake City, UT 84108 USA; 5 School of Public Health, University of Illinois in Chicago, 1603 W. Taylor St., 183, Chicago, Illinois 60608 USA

**Keywords:** Syrian, Refugee, War, Trauma, Common mental disorders, Family psychosocial support, Implementation, Resilience, Family intervention, Intervention development

## Abstract

**Background:**

This case study describes research, which is located in Turkey, where more than 750,000 Syrian refugees reside autonomously in Istanbul. The research developed and pilot tested a novel model for helping urban refugee families with limited to no access to evidence-based mental health services, by delivering a transdiagnostic family intervention for common mental disorders in health and non-health sector settings using a task-sharing approach. This case study addresses the following question: What challenges were encountered in developing and piloting a low intensity trans-diagnostic family support intervention in a humanitarian emergency setting?

**Discussion:**

The rapidly growing scale of humanitarian crises requires new response capabilities geared towards addressing populations with prolonged high vulnerability to mental health consequences and limited to no access to mental health, health, and social resources.

The research team faced multiple challenges in conducting this research in a humanitarian emergency setting including: 1) Non-existent or weak partnerships geared towards mental health research in a humanitarian emergency; 2) Lack of familiarity with task-sharing; 3).

Insufficient language and cultural competency; 3) Fit with families’ values and demands; 4) Hardships of urban refugees. Through the research process, the research team learned lessons concerning: 1) building a coalition of academic and humanitarian organization partners; 2) investing in the research capacity building of local researchers and partners; 3) working in a community-collaborative and multi-disciplinary approach.

**Conclusion:**

Conducting research in humanitarian emergency settings calls for innovative collaborative and multidisciplinary approaches to understanding and addressing many sociocultural, contextual, practical and scientific challenge.

## Background

The project was located in several neighborhoods in Istanbul, Turkey, where over 750,000 Syrian refugees are resettled. It developed and pilot tested a novel model for helping urban refugee families in settings with limited or no access to evidence-based mental health services for refugees. It delivered a transdiagnostic family intervention for symptoms of common mental disorders (CMD), such as depression, post-traumatic stress disorder (PTSD), and anxiety, in health sector and non-health sector settings using a task-sharing approach.

### Humanitarian context

The war in Syria displaced over 5.6 million persons to neighboring low and middle-income countries (LMIC). 3.6 million Syrian refugees live in Turkey, now the world’s largest refugee hosting country, with only about 2.85% living in refugee camps [1]. Syrian refugees are “guests” in Turkey as defined by the Temporary Protection Regulation, which assumes that most refugees will return to Syria once the prolonged conflict ends [2].

In Turkey, Syrian refugees have limited access to housing, work, information, food, education, and health and mental health care [3]. Syrians do low wage work with no benefits [4, 5]. Forty percent of school-aged children are not enrolled in Turkish schools [6] with child labor being a driving factor [7]. Differences in language and culture make integration a challenge [8, 9]. In Turkey, a 2017 poll indicated that 79% of respondents had an unfavorable view of Syrian refugees [10].

Syrian refugees are at high risk for CMD symptoms due to war trauma compounded by displacement stressors [11]. They experienced high rates of conflict-related violence and family loss and separation, followed by the daily stressors of displacement, such as lack of resources, discrimination, loss of social networks, limited livelihood options, and uncertainty about their future [12, 13]. Several prior studies assessed the mental health of Syrian refugees in Turkey and found rates of common mental disorders in adults between 15 and 42% [14–16]. Syrian refugee children also have high exposure to severe traumatic events and nearly half have PTSD symptoms [17]. Most Syrian refugees show little knowledge and awareness of mental illness, self-care strategies, or clinical treatment, and express high stigma towards mental health problems and services [18].

The scientific and professional mental health literature on Syrian refugees has largely looked through an individual lens and focused on PTSD. Despite the family orientation of Syrian culture, there was little evidence of family interventions being developed or deployed for the refugees. The researchers initially conducted longitudinal qualitative research with 30 Syrian refugee families so as to better characterize family stressors and coping mechanisms [19]. The researchers concluded that many families may be able to benefit from a family-focused intervention. This called for developing such interventions which could be conducted by refugee peer providers. This approach is also supported by prior research on the positive impact of family well-being, such as family resilience and family communication [20] on both adult and child family members, who each have unique risks and vulnerabilities in humanitarian emergencies [17].

### Research study

This project developed and pilot tested for feasibility an intervention referred to as Low Intensity Family Support (LIFS), a novel trans-diagnostic intervention for refugee families with common mental disorders, delivered in a multiple family group. This multiple family group is 6 to 8 families meeting together for four weekly two-hour group sessions at a community-based organization led by trained lay service providers. The sessions were designed to be led by lay persons who are Syrian refugees themselves. The sessions were held on weekends and took place in meeting rooms in the community that could accommodate 20 to 30 persons. Babysitting for younger children was provided so mothers could attend.

The LIFS model merged components from two theoretical frameworks underlying its two major intervention components. First, LIFS is based upon stress reduction as applied in the Problem Management Plus (PM+) intervention (described later) [20]. Second, LIFS is also based on family resilience, which explains family protective processes that can ameliorate the negative consequences of hardships and challenges and enable healing and growth in families facing adversity [21, 22]. The family resilience approach utilized came from the family support and education group model called Coffee and Families Education and Support (CAFES) which was implemented with Bosnian refugees in Chicago [23].

Each of the four sessions combined and adapted components from both PM+ and CAFES. These four sessions aim to mobilize multi-level individual and family coping strategies so as to address multiple outcomes of adults and children. Stress reduction strategies (e.g. slow breathing and grounding) aim to diminish CMD symptoms in family members. Family problem solving strategies aim to improve family communication and support. Psychoeducation strategies aim to increase family members’ knowledge and improve attitudes regarding mental health, especially related to trauma and PTSD. Linkage to care strategies aim to increase access to community-based mental health services for family members.

This research was designed to answer several research questions, which are needed to advance the field (see Table 1). To answer these questions, the research plan was organized around three specific aims. Aim 1 formed a Family Support Design Team (FSDT) to develop the low-intensity family support (LIFS) intervention for implementation in community sites using a four-session multiple family group format. Aim 2 piloted LIFS with families in community and clinical sites, and then through observations and qualitative interviews, assesses LIFS’ feasibility, fidelity, the impact of context and local capacity, the experiences of intervention delivery, and practitioner and organizational perspectives on scale up. Aim 3 conducted pre, immediate post, and 3-month post assessments of the refugee families who received LIFS in all sites, to demonstrate the kind of pre-post changes that have been reported for comparable interventions and to determine key parameters of interest with sufficient accuracy and precision.

**Table 1:** Developing and implementing family support research questions

Domain	Research Questions
**Intervention** (Aim 1)	How can we address CMD symptoms of both children and parents of displaced families and support protective family processes through a low intensity trans-diagnostic family support (FS) intervention?
**Uptake** (Aim 1 and Aim 2)	How can the group facilitators and community-based organizations best-overcome obstacles to promote engagement and retention in the LIFS groups?
**Intervention Acceptability** (Aim 2 & Aim 3)	Were the family members satisfied with the LIFS intervention content, delivery, and length? How did it fit with the sociocultural, environmental, and organizational contexts?
**Intervention Feasibility** (Aim 2 & Aim 3)	Was LIFS feasible as indicated by recruitment, attendance, retention, and fidelity, safety for participants and providers, and completion of outcome assessments?
**Implementation** (Aim 2 & Aim 3)	What do the families and facilitators think about offering LIFS to more refugee families? What are the opportunities or obstacles and how can they be best addressed to facilitate scale up?
**CMD Outcomes** (Aim 3)	Were there less depression, anxiety, and traumatic stress symptoms as a function of LIFS at 3-month assessments? Were these symptoms less after 3-months-post compared with immediate-post assessment?
**Family Outcomes** (Aim 3)	Was there improvement in family members’ knowledge and attitudes regarding responses to adversity, family support, and family problem solving, and accessing external resources as a function of LIFS at 3-month post assessments?
Constructs & Measures (Aim 3)	What constructs and measures are the best fits for evaluating a family support intervention in an LMIC?

In Istanbul, the researchers looked for collaborators with both scientific and practical experience in working with Syrian refugees and were eventually able to identify an academic medical center partner (Medeniyet University). This partnership also led the researchers to engage a new partner, the Turkish Red Crescent (TRC), as well as five local NGO partners (CAN, Hiraeth, Nour, Insan, and Yusra). These partners greatly strengthened the research team and have enabled the team to successfully develop and implement the project.

This research aimed to deliver an adapted and pilot tested low intensity family support model and preliminary pilot data that could inform the development of a follow-up larger scale study. The findings should also build knowledge on research approaches to building refugee services in LMIC’s and low resource settings in high-income countries (HICs).

## Discussion

### Scientific importance of the research

There is an urgent need for research to advance evidence-based brief psychosocial interventions that can be scaled up during humanitarian emergencies [24]. The World Health Organization’s Department of Mental Health and Substance Abuse, has been developing scalable psychosocial intervention to help address refugees’ mental health needs. They developed Problem Management Plus (PM+), which is a brief, basic, one-on-one, paraprofessional-delivered version for adults in communities affected by adversity [25–27].

In conflict-affected Pakistan, a pilot comparing PM+ to enhanced treatment as usual had high uptake, with 73% completing all sessions, and showed improvement in traumatic stress and functioning [28]. When WHO conducted a larger scale randomized controlled trial (RCT) of PM+ in Pakistan, it demonstrated clinically significant reductions in anxiety and depressive symptoms at 3 months among adults impaired by psychological distress [29].

Psychosocial interventions are needed for refugee families and humanitarian responses, both because parents and children are experiencing CMD symptoms, and because supporting family protective processes and learning self-care strategies can ameliorate the impact of CMD symptoms and stress caused by trauma and displacement. Syrian refugees, like many others, are strongly shaped by the family context. In a receiving country with limited resources for refugees, positive psychosocial outcomes for children and adults depend to a great extent on their families, yet refugee families find few empirically based services geared toward them [30, 31]. Refugee families often demonstrate resilience and strengths, but in highly adverse circumstances these processes can be strained or overwhelmed [32].

Based on the researcher’s community engagement with Syrian refugees and prior formative study of Syrian refugee families [19], the team concluded that a multiple family group approach previously used with Bosnian refugees in Chicago potentially fit well with Syrian culture. Of note, this type of family intervention is designed to include adolescents age 12 and above, with time allotted in each session for adolescents and adults to meet in separate breakout groups [33]. From our previous contact with CBO’s and mental health professionals in Turkey the available mental health interventions for addressing CMD’s were based on individual approaches.

Overall, the study aimed to build knowledge that encourages NGOs and governments to consider making family interventions more of a focus in humanitarian emergencies and beyond. In low- and middle-income countries which are not facing a humanitarian emergency, family interventions have been used with refugees and migrants [33–35]. Family interventions for refugees and migrants are also potentially helpful in low resource settings in HICs. For example, in the U.S. a large number of migrant families from Mexico and Latin America face high stress and CMD risks due to the threat of deportation on top of the ordinary exposure to adversity [36]. A family support intervention delivered in churches and community-based organizations, could help to strengthen their capacities for coping with adversity.

### Research methods

Aim 1 activities convened the Family Support Design Team (FSDT) and drafted the LIFS manual. Aim 2 partnered with the 6 community and clinical sites, trained the workers, having offered the intervention to 72 families, and then conducted observations and qualitative interviews to assess implementation issues. Aim 3 conduced pre, immediate post, and 3-month post assessments of the 72 families who received the intervention.

Regarding Aim 3, the primary hypothesis was that LIFS participants would report fewer symptoms of depression, anxiety, and traumatic stress from baseline to immediate post and 3-month post. Our secondary hypothesis was that LIFS participants would report improved knowledge and attitudes regarding responses to adversity, family support, family problem solving, and accessing external resources, and that these would explain improvements in CMD symptoms. In addition, the team also studied engagement and retention of families in LIFS, both as outcomes and as possible determinants of the primary outcomes. The researchers plan to conduct analyses: 1) to demonstrate the feasibility of the implementation and evaluation methods; 2) to explore patterns of attendance and retention to LIFS groups, to inform the researchers in making modifications; 3) to demonstrate the kind of pre-post changes that have been reported for comparable interventions, for both primary participants and family members; 4) to determine key parameters of interest with sufficient accuracy and precision. The data collection for Aims 2 and 3 started in December 2019.

### Challenges to the research

The research team faced multiple challenges in conducting this research in a humanitarian emergency setting, even beyond those challenges generally encountered in LMICs or low-resource settings. These challenges and the approaches to them are summarized below.

#### Non-existent or weak partnerships geared towards mental Health Research in a humanitarian emergency

The researchers were able to identify both academic centers and humanitarian organizations, but it was difficult to find academic centers with existing partnerships with humanitarian organizations, or vice versa. Syrian refugees were generally not a focus of interest for service or research by academic medical centers in Istanbul. It was also difficult to identify local academic partners who had research capacity in mental health and implementation science. The local NGO partners were experienced in working with refugees but, had little research experience. The Turkish Red Crescent had an Academy unit and was interested in building mental health and implementation science research capacity.

#### Lack of familiarity with task-sharing

It was challenging to find a psychiatrist who would accept the task-sharing approach, which aimed to deliver interventions that did not depend upon specialist service providers. The U.S. and Kosovar investigators were able to identify a child and adolescent psychiatrist, who was a certified trainer in cognitive behavioral therapy (CBT) and had conducted research with Syrian adolescent refugees in school settings in Istanbul [37, 38]. He was first identified through a literature review on refugee mental health in Turkey, then was contacted and after multiple meetings, eventually joined the project. He became a co-investigator and was highly involved in designing the research plan and building the LIFS model.

#### Insufficient language and cultural competency

Both the U.S. and Kosovar investigators and the local Turkish investigators involved in the research faced limitations of language and cultural competency regarding Syrian refugees. Two Syrians familiar with the community and NGOs were hired as the project manager and assistant and they helped the research team to overcome these hurdles. The Syrian project personnel were identified with the help of the Turkish psychiatrist who had worked with them in previous psychosocial projects. To address cultural and contextual challenges, the research team also reviewed the literature on cultural issues related to mental health and service delivery for Syrian refugees. Some key issues which emerged involved the importance of faith and religion in coping, high levels of stigma regarding mental health, and gender roles and perspectives.

#### Fit with families’ values and demands

One of the major challenges faced was planning for a family intervention that would work with families who had cultural values and practices around gender relations that were traditional and appeared inflexible in terms of women and men interacting and sharing the same space. A second challenge was involving fathers in all the sessions as they were either working or having other obligations. Last but not least, another challenge was the families’ high stigma and little knowledge regarding mental illness and mental health help seeking.

#### Hardships of urban refugees

Living on their own in Istanbul, these families faced many burdens and high demands on their time especially related to long working hours, working during weekends, and child labor. Just prior to the time of implementation in October 2019, the Turkish government began to more strictly enforce policies regarding the residential permits of Syrian refugees. When refugees came to Turkey, they were allowed to live in a particular region where they were registered, but could not move to another, such as Istanbul, to get work. This stricter enforcement with possible deportation to the city where they were registered or to Syria, caused fear among refugees. The research team did not face any challenges from any federal or municipal governmental agencies in the implementation process.

### Research strategies

To address the above challenges, the research team and partners tried to develop a shared understanding and problem-solving strategies through ongoing discussions. This demanded a great deal of flexibility and patience from all. At several junctures, to properly deal with these issues, the research process was slowed down, but then eventually resumed. Addressing the unique challenges faced by the research team, required specific strategies, summarized below.

#### Coalition building

The researchers built a coalition of academic and humanitarian organization partners and worked on strengthening relations between them. This coalition included an academic medical partner, six local community-based organizations working with Syrian refugees and the Turkish Red Crescent, one of the major actors in Turkey in the provision of mental health and psychosocial services to Syrian refugees.

#### Building research capacity of local partners

The researchers invested in building the research capacity building of the local researchers and partners. This included the research capacity of several faculty members at Medeniyet University, members of the Academic Unit of the TRC, who had some research experience, and workers from the NGOs, who had none.

#### Engaging refugee family experiences and perspectives

The researchers convened the Family Support Design Team (FSDT) to draw upon refugee family experiences and perspectives so as to adapt the existing interventions and design new components. The FSDT first met face-to-face for 3 days in month two in Istanbul, followed by regular Skype calls and regular emails, and a follow-up three-day meeting in month 6, so as to complete drafting the LIFS manual [39]. The FSDT was co-led by the researchers, and included other LMIC researchers as well as eight additional refugee peer providers and health care workers. The overall aim was to build a model that was brief and simple enough to be delivered by community workers and nurses. Draft materials were prepared and distributed in advance, in English and Arabic and decisions were made by consensus after deliberation.

#### Flexible intervention design

The research team designed a flexible structure for the intervention to accommodate gender considerations including use of the Internet and mobile technology. For example, to accommodate families who preferred to sit all together rather than to mix with other families LIFS provided seating arrangements in which families were seated at separate tables as in a restaurant. In addition, each group had one male and female facilitator where female facilitators reinforced women’s participation given concerns that women would not speak out in mixed groups or in the presence of their husbands. Furthermore, the team produced easily accessible videos such as stress reduction exercises to be used by members who might have not attended the session and as a reminder of exercises to be done at home.

#### Fostering evidence-based policy and program development for refugees

In addition to addressing the three research aims, the researchers and the TRC collaboratively developed and implemented an additional plan to foster evidence-based policy and program development in mental health for refugees by expanding communication and collaboration between the researchers and end users, including the TRC and other NGOs, including practitioners, managers, and policy makers. The additional activities were convening a refugee mental health implementation research group composed of researchers and end users in the TRC that collaboratively: a) conducted trainings on the state-of-the-science in implementation science research (e.g. strategies for implementation, dissemination, and evaluation of effective mental health interventions); b) disseminated research findings on proven mental health assessments and interventions; and c) built a model for best impacting the TRC organizational structure, climate, culture, and processes regarding the implementation, dissemination, and evaluation of effective and evidence based mental health interventions.

## Conclusions

Through the research process, the team learned several key lessons regarding conducting research in a humanitarian emergency setting. One, it is helpful to build a coalition of academic and humanitarian organization partners who can ensure that the research is focused on issues that matter for service providers and recipients. Two, it is helpful to invest in mental health research capacity building of the local partners to strengthen research implementation and facilitate dissemination and scaling up. Three, utilizing a community-collaborative and multi-disciplinary approach can help to best understand and address multiple key sociocultural, contextual, and scientific challenges needed to develop, adapt and implement the LIFS model.

## Data Availability

The current article has no data on which the findings are based except personal notes of authors.

## Abbreviations

CBT: Cognitive Behavioral Therapy
CMD: Common Mental Disorders
FSDT: Family Support Design Team
LIFS: Low Intensity Family Support
LMICs: Low- and Middle-Income Countries
PM +: Problem Management Plus
RCT: Randomized Clinical Trials
TRC: Turkish Red Crescent
U.S.: United States
WHO: World Health Organization
